# From Virtual to In-Person Teaching After COVID – Face-to-Face Simulation Gives Greater Improvement in Confidence and Satisfaction

**DOI:** 10.1192/bjo.2024.290

**Published:** 2024-08-01

**Authors:** Daniel Di Francesco, Alexandra Colombo-Sansom

**Affiliations:** 1 Sussex Partnership NHS Foundation Trust, Hailsham, United Kingdom; 2 Sussex Partnership NHS Foundation Trust, Brighton, United Kingdom

## Abstract

**Aims:**

To adapt a virtual simulation training session for junior doctors, developed during COVID, to an in-person format.

To compare self-reported differences in knowledge and usefulness of the session across the two formats.

**Methods:**

Initially a virtual simulation training session was developed and run in the induction program for junior doctors rotating onto psychiatry. This involved a series of 10-minute simulations tackling 5 emergency scenarios:

Using section 5(2); Acutely agitated patient and rapid tranquilisation; Neuroleptic malignant syndrome; Alcohol withdrawal and detoxification; ligature injury.

Written briefs were constructed and standardised actors delivered the content through a video call with the doctors. A facilitator was able to provide key data, including NEWS scores and exam findings. This was followed by a ten minute debrief, giving feedback on communication, and discussion around the key learning points.

After COVID restrictions were eased, this programme was adapted to a face-to-face format. New, Trust-specific, resources were developed – paper NEWS charts, drug charts, alcohol detoxification pro-forma, and section 5(2) paperwork, which were made available to the candidate during the scenario.

Self-reported scores were collected in the virtual (N = 117) and face-to-face (N = 19) sessions across several domains: in the usefulness and relevance, improvement in knowledge, and overall benefit of the teaching programme, as well as free-text feedback.

**Results:**

Scores were collected on a 5-point Likert scale, (from 1 - strongly disagree, to 5 - strongly agree) and a mean score was calculated, and p value calculated with a two-tailed Mann Whitney U test. The scores showed improved ratings in the face-to-face sessions across all domains - improvement in knowledge (from 4.2 to 4.6; p = 0.0005), and overall satisfaction (from 4.18 to 4.63; p = 0.00036), usefulness and relevance (from 4.06 to 4.68; p = 0.053, though this last domain did not reach statistical significance).

Free text feedback highlighted the positive aspects of the pacing, organisation and delivery of feedback from actors and facilitators.

There were also suggestions for improvement - to adapt the scenarios to better capture the wide variation in doctors' previous experience of psychiatry, and to reduce the group sizes.

**Conclusion:**

A simulation teaching session developed during COVID was successfully transitioned to a face-to-face format. This allowed a higher-fidelity environment with trust specific scenario materials and enabled more realistic communication with the actors. The face-to-face session was found to deliver higher improvement in self-reported knowledge and satisfaction, compared with the virtual session.

